# Supplementary material for: Single-cell characterization of leukemic and non-leukemic immune repertoires in CD8+ T-cell large granular lymphocytic leukemia
Source: Nat Commun. 2022 Apr 11;13:1981. doi: 10.1038/s41467-022-29173-z (PMC9001660; doi:10.1038/s41467-022-29173-z)
Supplement: Supplementary file 1 — Supplementary Information [file 41467_2022_29173_MOESM1_ESM.pdf]

Supplementary Figure 1

a

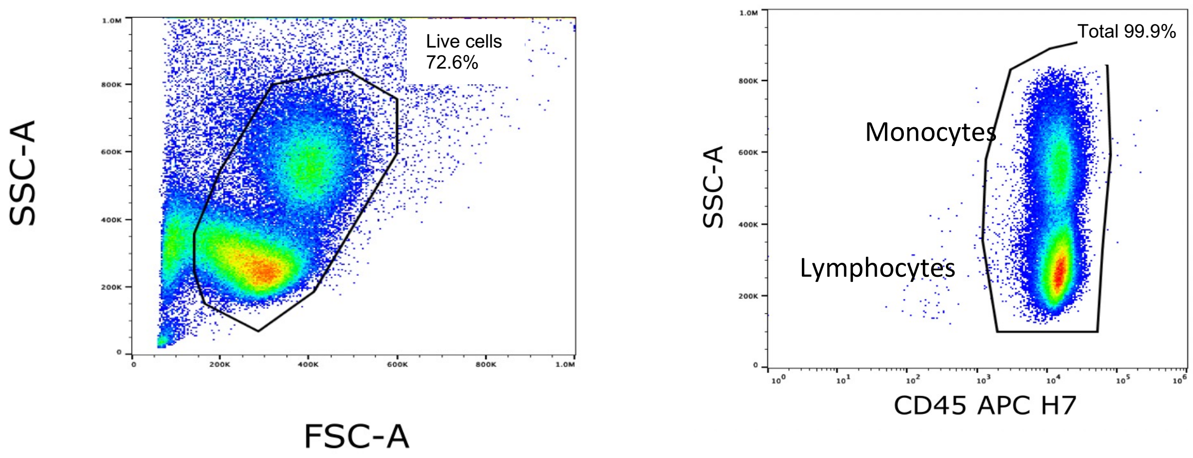

Supplementary Figure 1: Cell sorting strategy for scRNA+TCRαβ-seq

a) Representative gating strategy for the CD45+ sorted cells in the scRNA+TCRαβ-seq cohort from peripheral blood mononuclear cells with fluorescent activated cell sorting based on side-scatter (SSC), forward-scatter (FSC), and CD45 (APC-H7).

Supplementary Figure 2

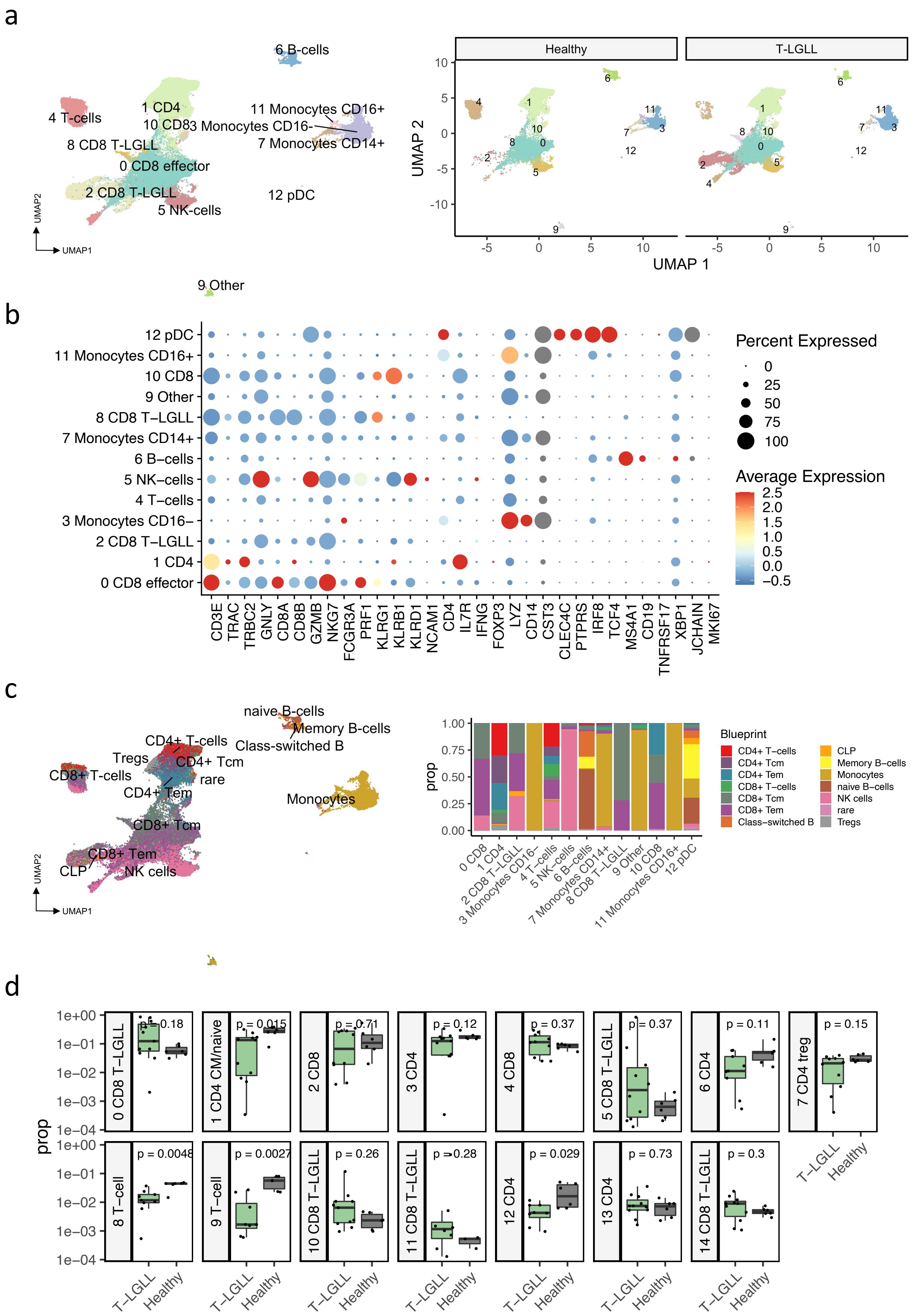

Supplementary Figure 2: Annotation of CD45+ sorted cells from T-LGLL and healthy

a) Left: UMAP representation of CD45+ sorted cells from 11 T-LGLL and 6 healthy donor samples profiled with scRNA+TCRαβ-seq, as shown in Fig. 1b. Different colors indicate clusters. Right: Cells from healthy controls and T-LGLL patients plotted separately. **b)** Expression of canonical markers used to annotate the clusters. The dot size correlates with the number of cells expressing a given gene while the color denotes the gene expression as Z-values. **c)** Left: The same UMAP presentation as in panel A, where the colors correspond to the prediction results with automated reference-based method SingleR, where Blueprint was used as a reference. Right: the same prediction results per cluster. **d)** Cluster abundancies in T-LGLL patients and healthy controls. *P*-values were calculated with two-sided Mann-Whitney test.

Supplementary Figure 3

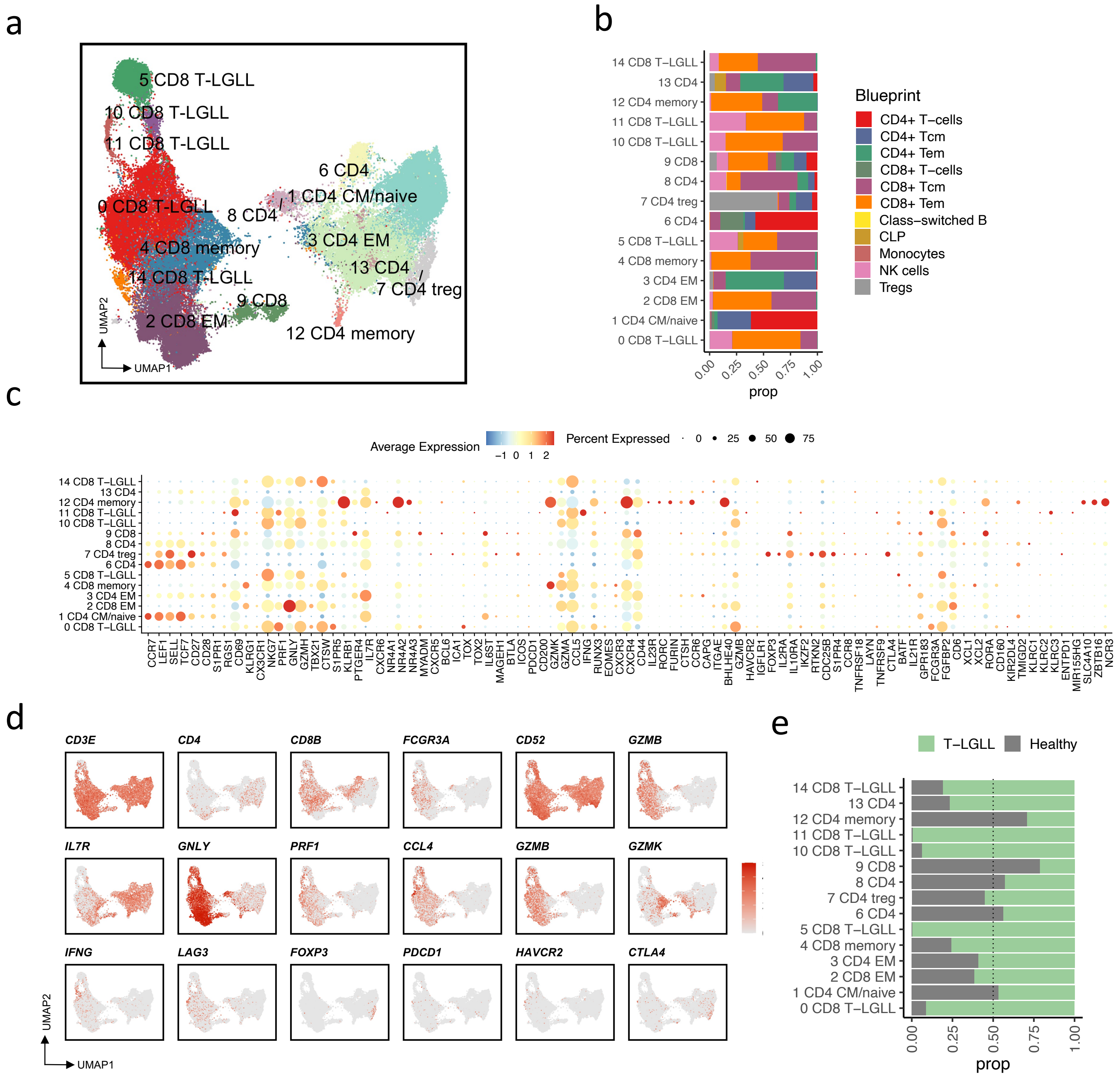

Supplementary Figure 3: Annotation of cells with detected TCR from T-LGLL and healthy

a) The same focused UMAP representation as in Fig. 1c of the cells with detected TCR from 11 T-LGLL and 6 healthy donor samples. **b)** Prediction results with automated reference-based method SingleR, where Blueprint was used as a reference per cluster. **c)** Expression of canonical markers used to annotate the clusters. The dot size correlates with the number of cells expressing a given gene while the color denotes the gene expression as Z-values. **d)** Scaled expression of selected genes highlighted in the same UMAP representation as in panel A. **e)** The abundances of cells from T-LGLL and healthy in different clusters.

Supplementary Figure 4

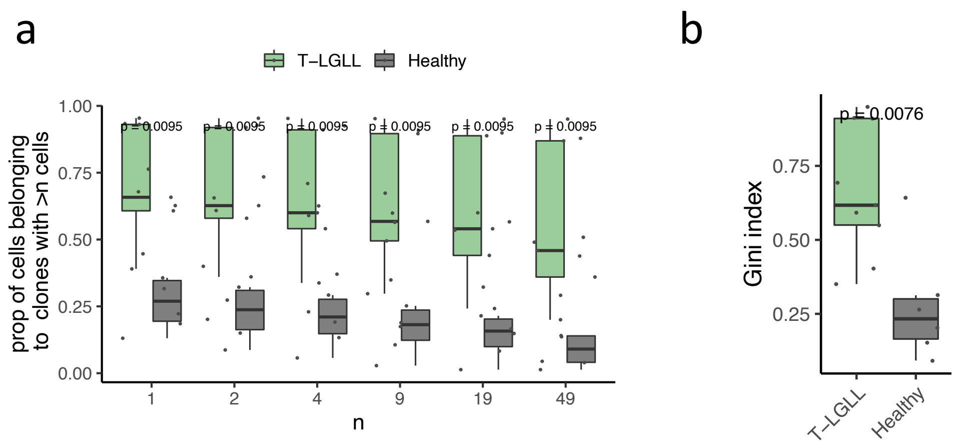

Supplementary Figure 4: Clonality of cells with detected TCR from T-LGLL and healthy

a) Box plot showing the amount of cells for expanded clonotypes with different thresholds for clonotype size. *P*-values were calculated with two-sided Mann-Whitney test. **b)** Clonality index (Gini, higher denotes more clonal) between T-LGLL and healthy. *P*-values were calculated with two-sided Mann-Whitney test.

Supplementary Figure 5

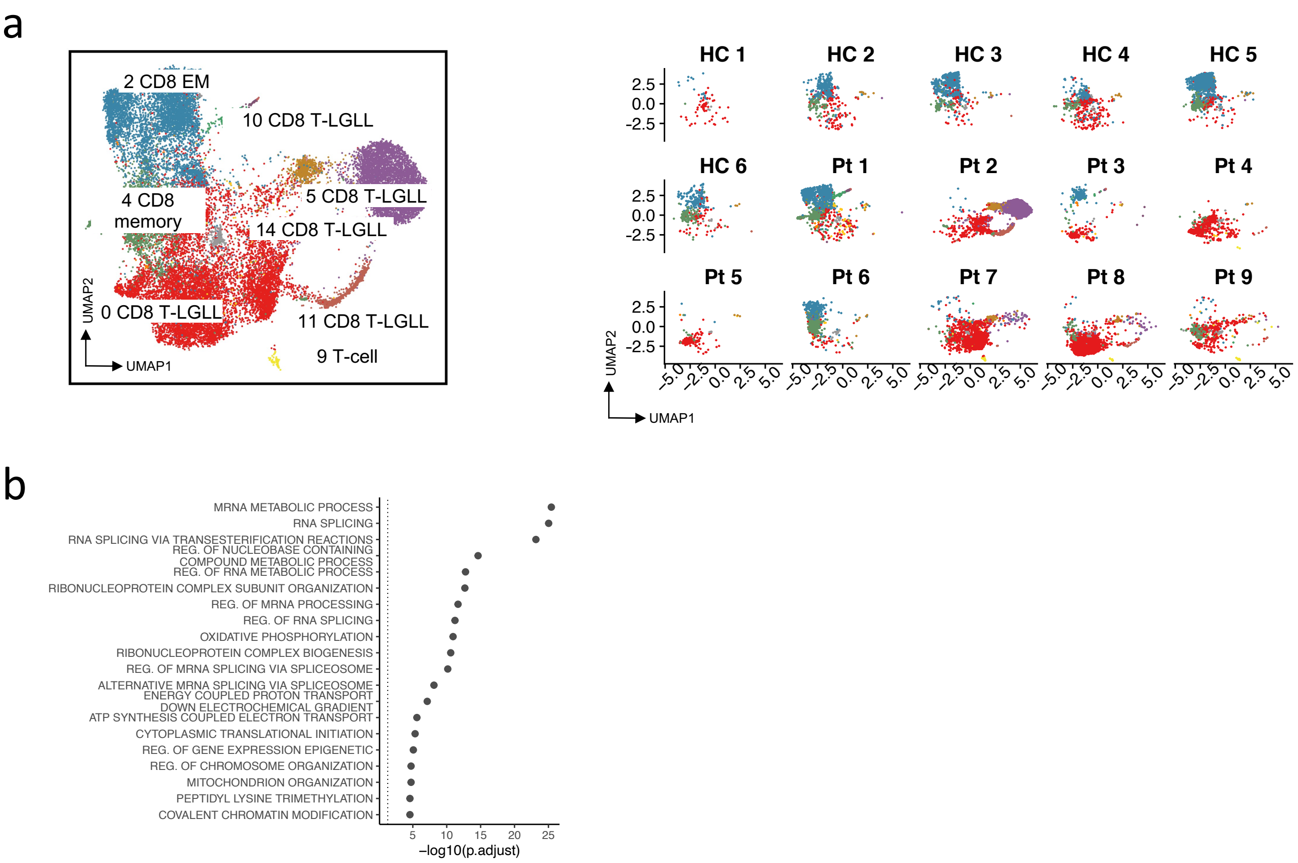

Supplementary Figure 5: Hyperexpanded clonotypes in T-LGLL vs healthy

a) Left: The same focused UMAP as in Fig. 1e of cells with TCR where hyperexpanded clonotypes (TCR detected at least 10 times) are highlighted. Right: The distribution of cells from T-LGLL and healthy samples are shown. **b)** Upregulated GO-pathways (P_{adj}<0.05, Benjamini-Hochberg corrected Fisher's one-sided exact test on differentially expressed genes) in hyperexpanded clonotypes from healthy.

Supplementary Figure 6

a

b

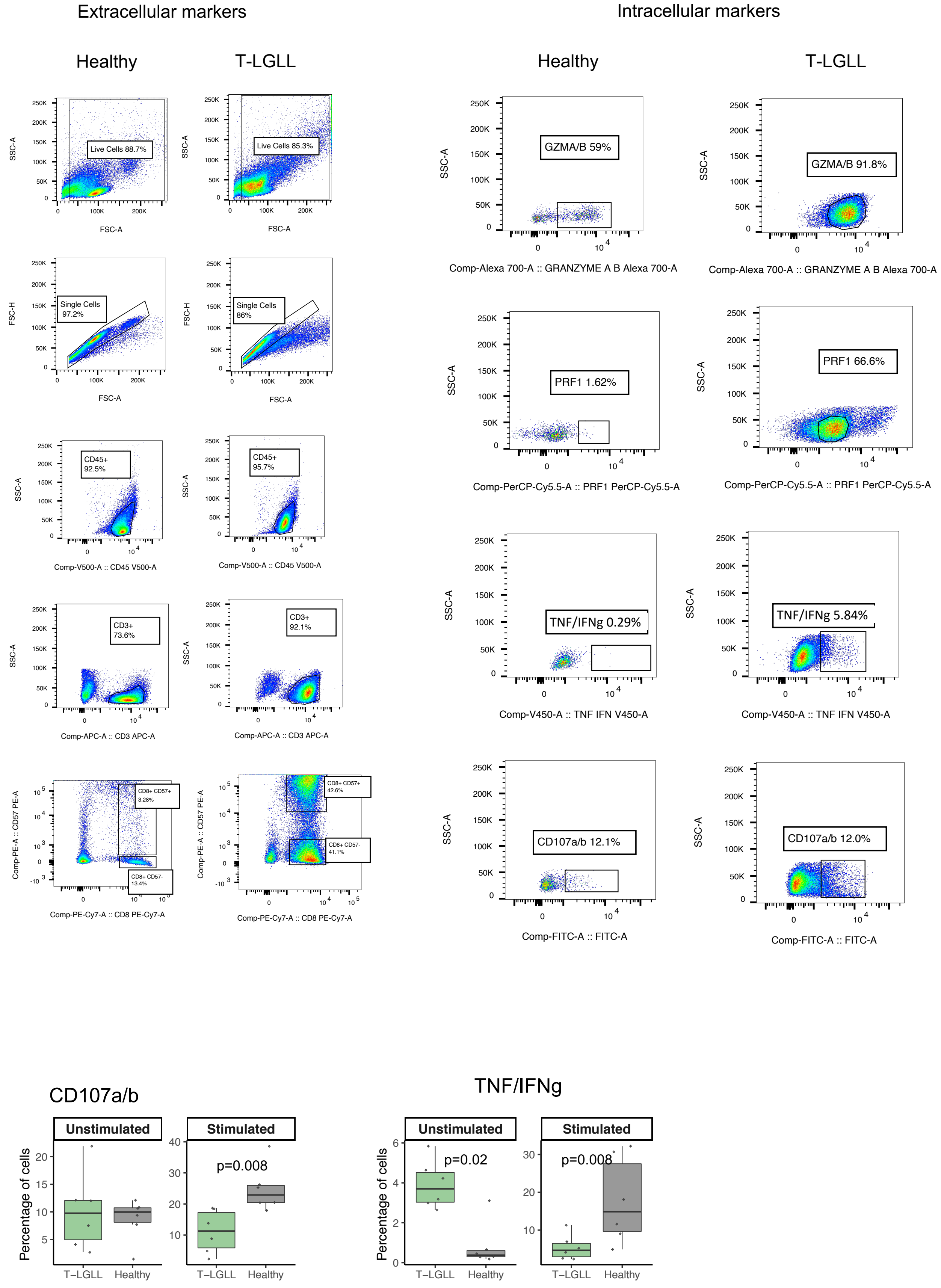

Supplementary Figure 6: Cell sorting strategy for detection of cytotoxic proteins in flow cytometry validation cohort and degranulation and cytokine secretion of T-LGLL cells

a) Representative gating strategy of extracellular (CD45 [V500], CD3 [APC], CD8 [PE-Cy7], and CD57 [PE]) and intracellular (GZMA/B [Alexa-700], PRF1 [PerCP-Cy5.5], TNF/IFNγ [V450], and CD107a/b [FITC-A]) markers in validation cohort profiled with flow cytometry. **b)** The percentages of CD107a/b+ or TNF/IFNg+ CD8+ cells under unstimulated and TCR stimulated (CD3, CD28 and CD49) conditions in the flow cytometry cohort. *P*-values were calculated with two-sided Mann-Whitney test.

Supplementary Figure 7

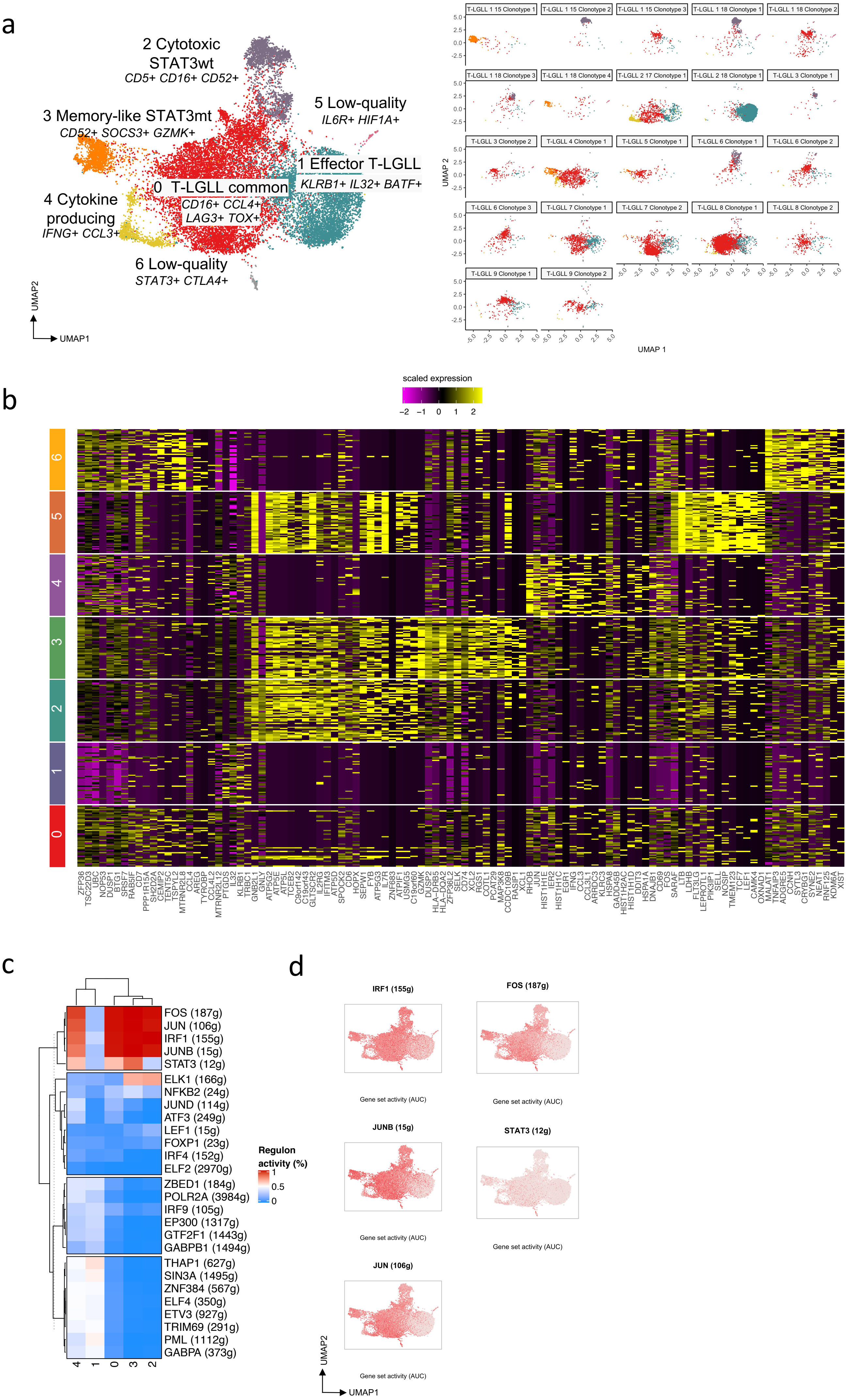

Supplementary Figure 7: Annotation of T-LGLL clonotypes' phenotypes

a) Left: The same UMAP representation as in Fig. 2b of inferred T-LGLL clonotypes from 11 T-LGLL samples. Right: The distribution of cells from T-LGLL clonotypes are shown. **b**) Top differentially expressed genes (P_{adj}<0.05, Bonferroni corrected t-test) between the T-LGLL clonotypes' phenotype clusters. **c)** Regulon activities identified with SCENIC in different T-LGLL clonotypes' phenotype clusters (clusters 0-4). **d)** Scaled expression of gene set activity of SCENIC identified regulons as areas under the curve (AUC) in the same UMAP representation as in panel A.

Supplementary Figure 8

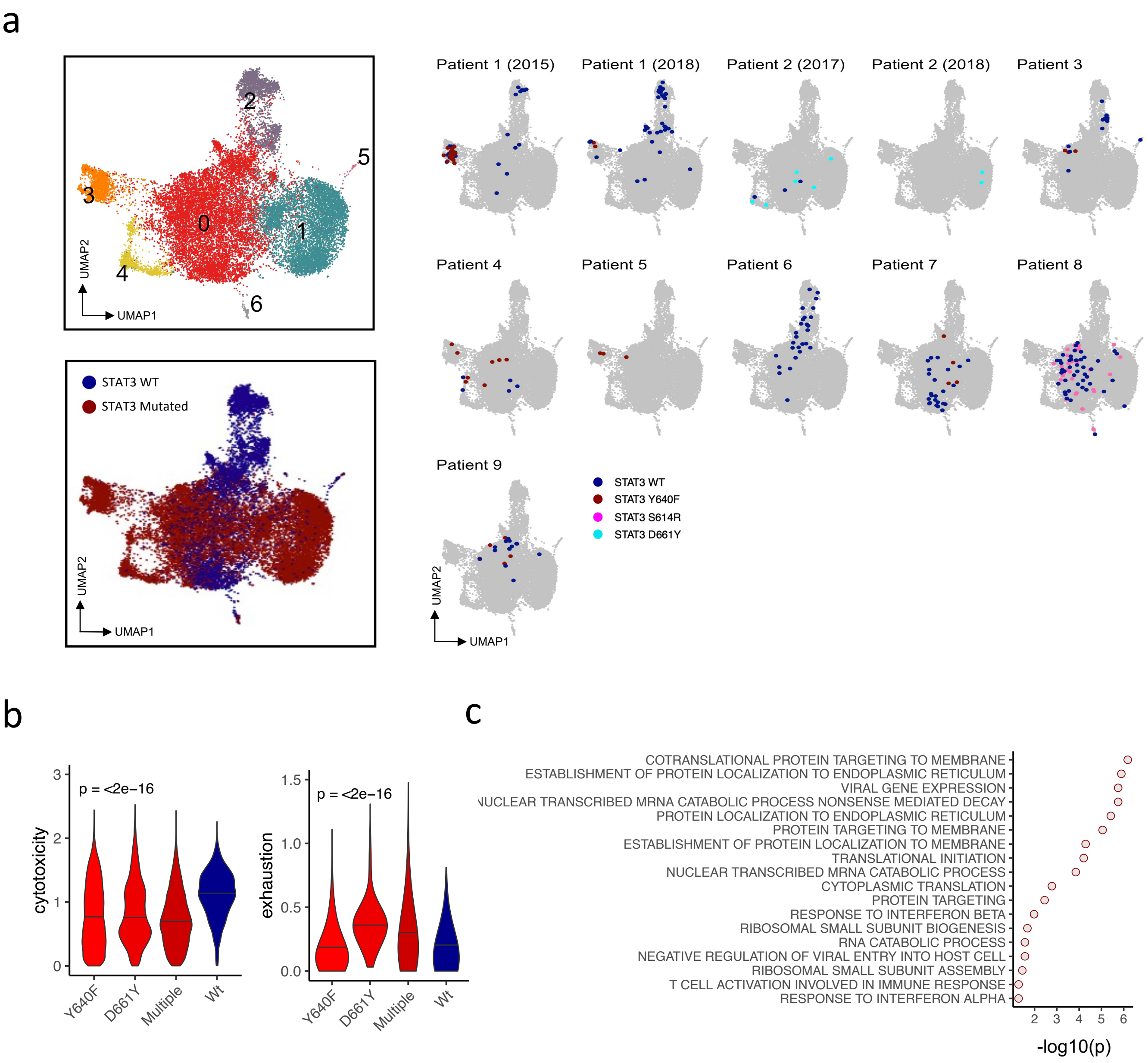

Supplementary Figure 8: Phenotypic differences between *STAT3* mutated and wild type T-LGLL cells in scRNA+TCRαβ-seq

a) Left upper panel: The same UMAP representation as in Fig. 2b of the transcriptomes of the selected 18 T-LGLL clonotypes as shown. Left lower panel: Imputed *STAT3* mutation status highlighted in the UMAP representation. Right: Detected *STAT3* mutation status received by variant detection from scRNAseq reads with Vartrix in individual patients in the same UMAP presentation as in Fig. 2b. **b)** Cytotoxicity and exhaustion scores of T-LGLL cells carrying different *STAT3* mutations. *P*-values were calculated with two-sided Mann-Whitney test. **c)** Top upregulated GO-pathways (*P*<0.05, unadjusted Fisher's one-sided exact test on differentially expressed genes) in *STAT3* mutated clonotypes in comparison to *STAT3* wild type clonotypes.

Supplementary Figure 9

a

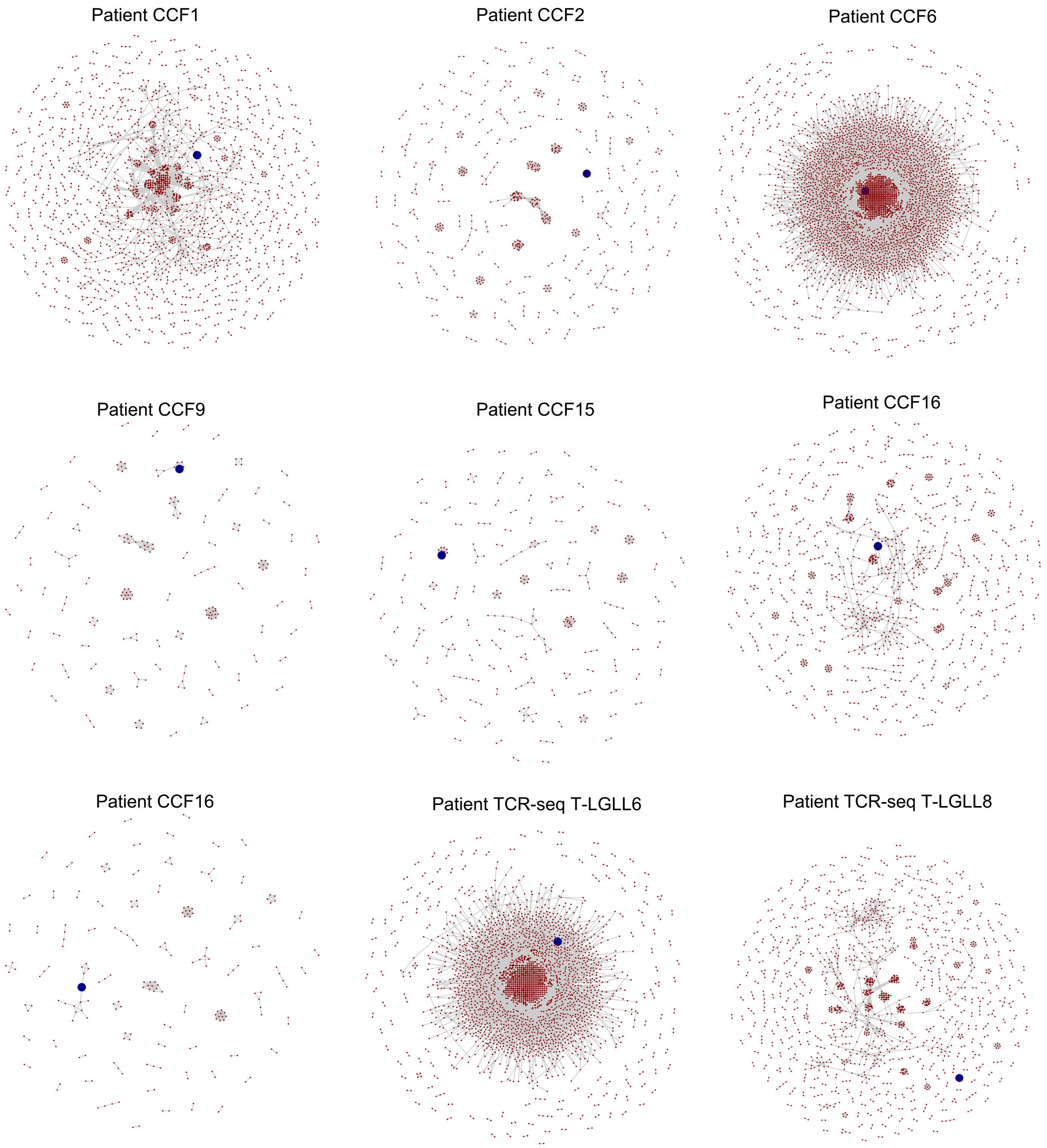

Supplementary Figure 9: Antigen drive in T-LGLL

a) Network plots showing antigen-driven clonotypes from nine selected patients with T-LGLL. Antigen drive denotes that T-LGLL clone shares amino acid-level similarities with its non-leukemic repertoire. Each dot (a vertice), is a TCR clonotype, and clonotypes with shared amino acid-level similarities are connected by a line (an edge). The T-LGLL clones are highlighted with a blue color and non-leukemic with a red color.

Supplementary Figure 10

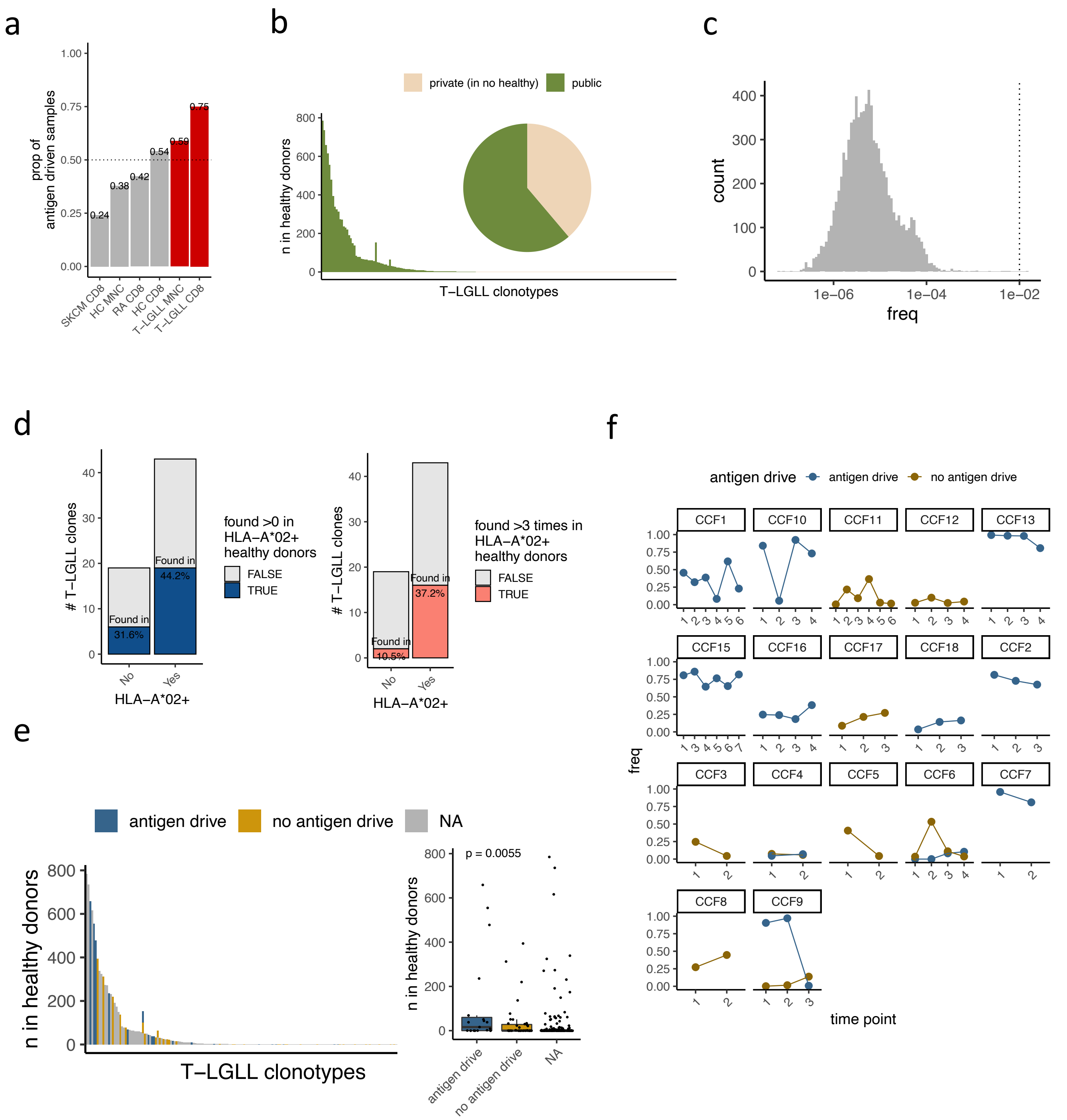

Supplementary Figure 10: The antigen drive and the amount of public clonotypes in T-LGLL

a) Presence of antigen-drive (i.e., whether the largest clonotypes have shared amino acid-level similarities with the rest of the TCR repertoire) in T-LGLL (mononuclear cell [MNC]-sorted *n*=17, CD8+-sorted *n*=10), metastatic melanoma sampled from blood (SKCM, *n*=29), rheumatoid arthritis (RA, *n*=32), and healthy controls (HC, MNC-sorted *n*=785, CD8+-sorted *n*=38). T-LGLL patients had more antigen-driven cases than the rest of the conditions (P<0.05, Fisher's one-sided exact test). All the non-leukemic samples were downsampled to the same read-depth (30,000 reads per sample). The results where downsampling was done on all the reads is shown in Fig. 4a. **b)** Number of times and proportion of TCRs from T-LGLL clonotypes found in healthy donors' (n=785) TCR repertoire. **c)** Histogram showing the frequencies TCRs from T-LGLL clonotypes found in healthy donors' TCR repertoire. Dashed line denotes frequency of 0.01. **d)** The number and percentage of 43 T-LGLL clonotypes from HLA-A*02+ patients and 1+ T-LGLL clonotypes from HLA-A*02 negative patients found in 294 HLA-A*02+ healthy donors. **e)** Number of times antigen-driven and not antigen-driven T-LGLL clonotypes were found in healthy donors' (*n*=785) TCRβ repertoires. NA: T-LGLL clonotype with unknown antigen drive status. P-values were calculated with two-sided Mann-Whitney test. **f)** Evolution of T-LGLL antigen-driven and no antigen-driven clonotypes in multiple time points.

Supplementary Figure 11

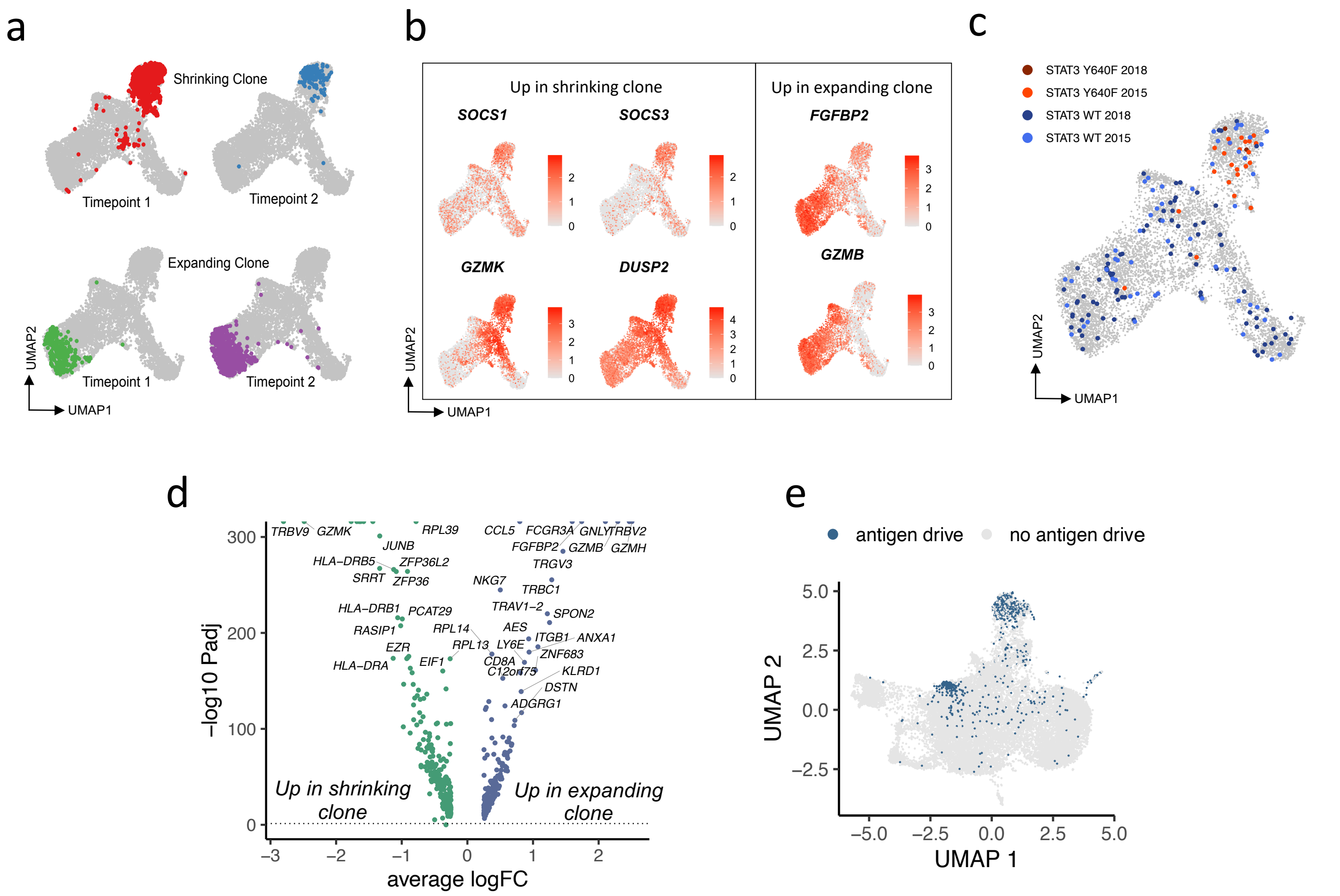

Supplementary Figure 11: Annotation of clusters in CD8+ cells from patient 1 with antigen-driven clonal drift

a) The same UMAP representation as in Fig. 4f of CD8+ cells from Patient 1 with clonal drift, where mutated shrinking and wild-type expanding *STAT3* cells with different TCRαβ are highlighted. **b)** Scaled expression of selected genes showed in the same UMAP as in panel A. **c)** The results from the Vartrix, where the mutated *STAT3* and wild-type *STAT3* cells are highlighted. **d)** Differentially expressed genes between (P_{adj}<0.05, Bonferroni corrected two-sided t-test) the expanding, antigen-driven *STAT3* wildtype T-LGLL clone and the non-antigen driven shrinking *STAT3* mutated clone. **e)** The same UMAP representation of T-LGLL cells as in Fig. 2b, where the antigen-driven cells from patient 1 are highlighted.

Supplementary Figure 12

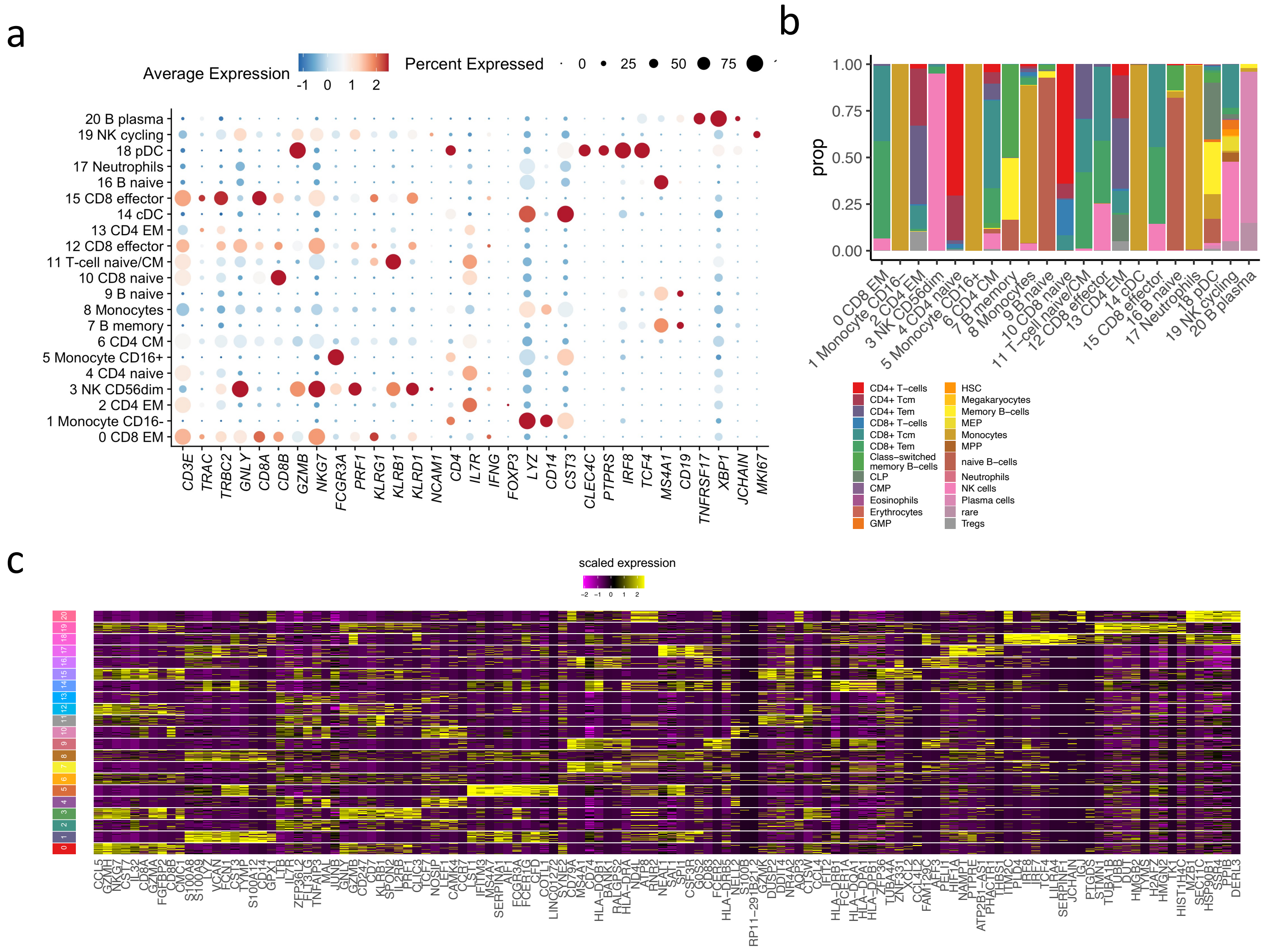

Supplementary Figure 12: Annotation of non-leukemic clusters in pan-cancer analysis

a) Expression of canonical markers used to annotate the clusters. The dot size correlates with the number of cells expressing a given gene while the color denotes the gene expression as Z-values. **b)** Prediction results with automated reference-based method SingleR, where Blueprint was used as a reference. **c)** Top differentially expressed genes (P_{adj}<0.05, Bonferroni corrected t-test) between clusters.

Supplementary Figure 13

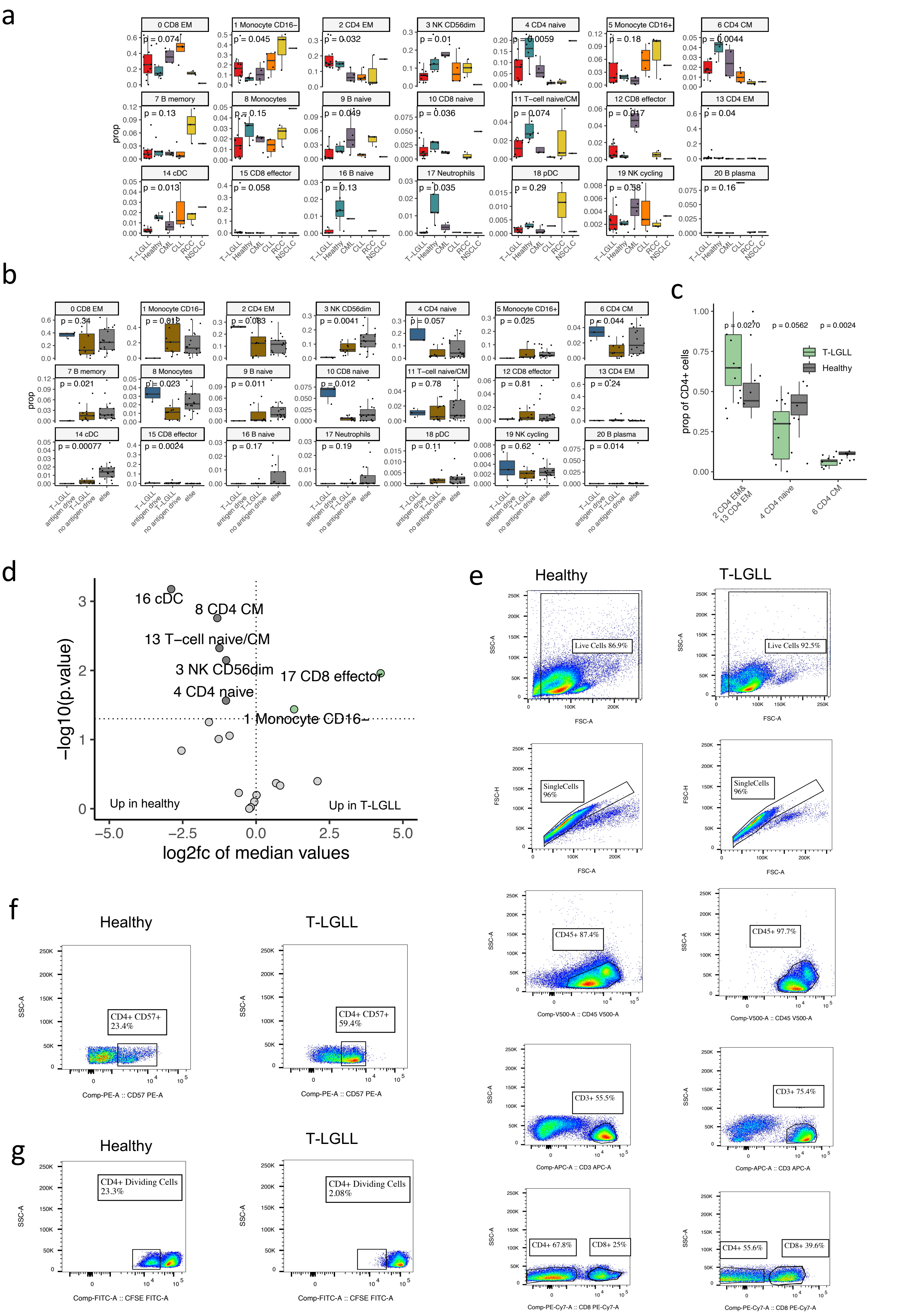

Supplementary Figure 13: Non-leukemic cluster abundances in pan-cancer analysis

a) Cluster proportions of non-malignant CD45+ sorted cells from 11 T-LGLL, 6 healthy donor, 4 CML, 4 CLL, 2 RCC, and 1 NSCLC samples profiled from peripheral blood with 10X technologies. *P*-values were calculated with two-sided Kruskal-Wallis test. **b)** Same as in panel A but where T-LGLL samples are divided by the presence of antigen drive (i.e., whether the largest clonotypes have shared amino acid-level similarities with the rest of the TCR repertoire) and the other profiles are pooled as "else", including 6 healthy donor, 4 CML, 4 CLL, 2 RCC, and 1 NSCLC samples. *P*-values were calculated with two-sided Kruskal-Wallis test. **c)** Proportion of different CD4+ subpopulations out of total CD4+ cells between patients with T-LGLL (n=9) and healthy (n=6). *P*-values were calculated with two-sided Mann-Whitney test. **d)** Differentially abundant clusters between patients with T-LGLL (n=9) and healthy donors (n=6). The horizontal line indicates P=0.05, as calculated with two-sided Mann-Whitney test. **e)** Representative gating strategy for CD4+ cells with markers CD45 (V500), CD3 (APC), and CD8 (PE-Cy7), in the validation cohort profiled with flow cytometry. **f)** Further representative gating strategy for CD4+CD57+ cells with CD57 (PE) in the validation cohort profiled with flow cytometry. **g)** Further representative gating strategy for proliferative CD4+ cells as measured by CFSE fluorescence (FITC) in the validation cohort profiled with flow cytometry.

Supplementary Figure 14

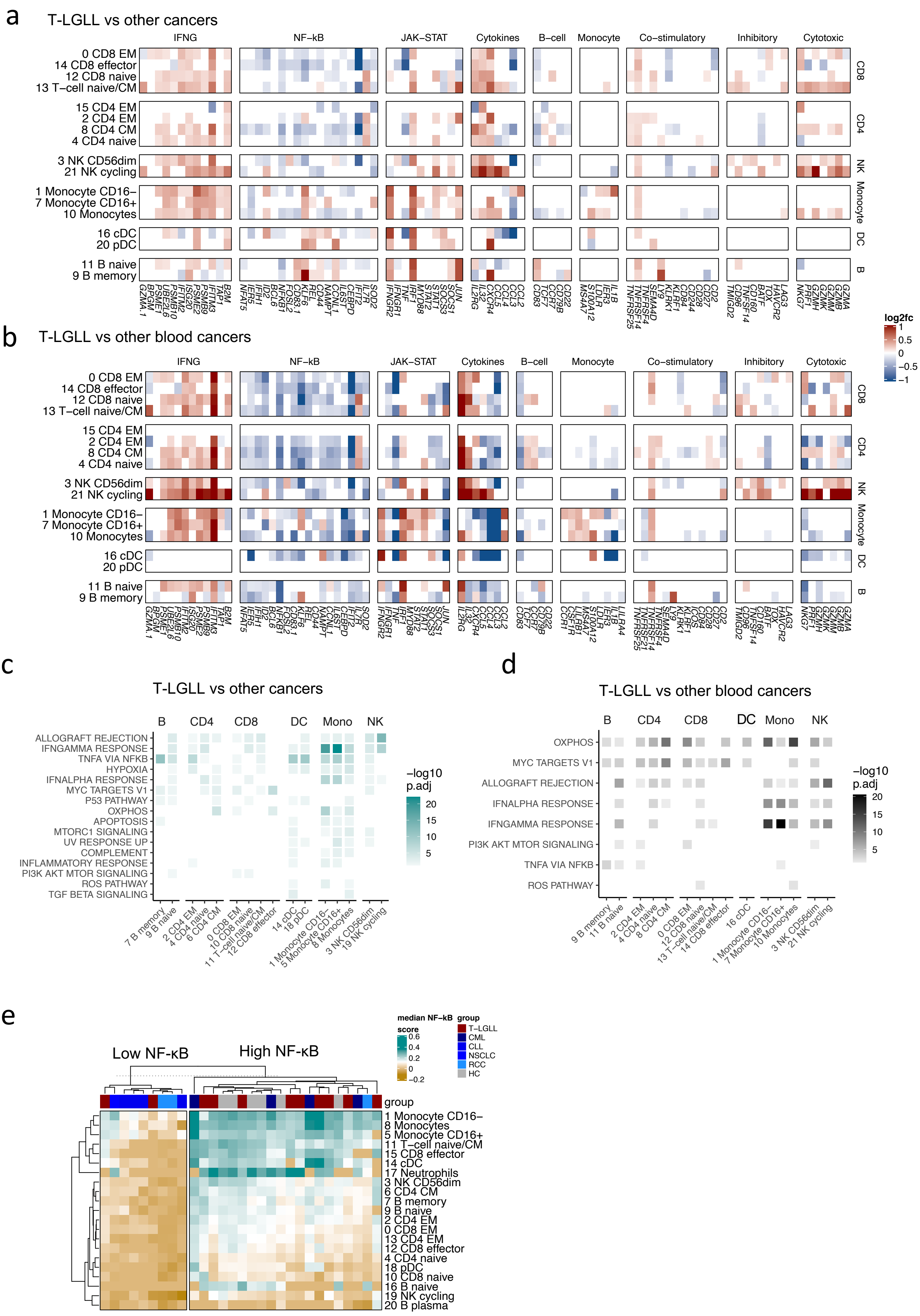

Supplementary Figure 14: Differentially expressed genes in non-leukemic clusters in pan-cancer analysis

a) Expression of selected differentially expressed genes between non-leukemic immune cells from patients with T-LGLL and patients with other cancers (CLL *n*=4, CML *n*=4, NSCLC *n*=1, RCC *n*=2). Values are presented as log2 fold-change (log2fc). **b)** Similar plot as in panel A but from patients with T-LGLL and patients with other blood cancers (CLL, CML). **c)** Upregulated HALLMARK-pathways (P_{adj}<0.05, Benjamini-Hochberg corrected one-sided Fisher's exact test on differentially expressed genes) in non-leukemic cells from T-LGLL in comparison to other cancers. **d)** Similar plot as in panel C but from patients with T-LGLL and patients with other blood cancers (CLL, CML). **e)** The median expression of Nf-κB response module score in different immune subsets in patients with T-LGLL, healthy, and patients with other cancers. Clustering was performed with Ward's linkage.

Supplementary Figure 15

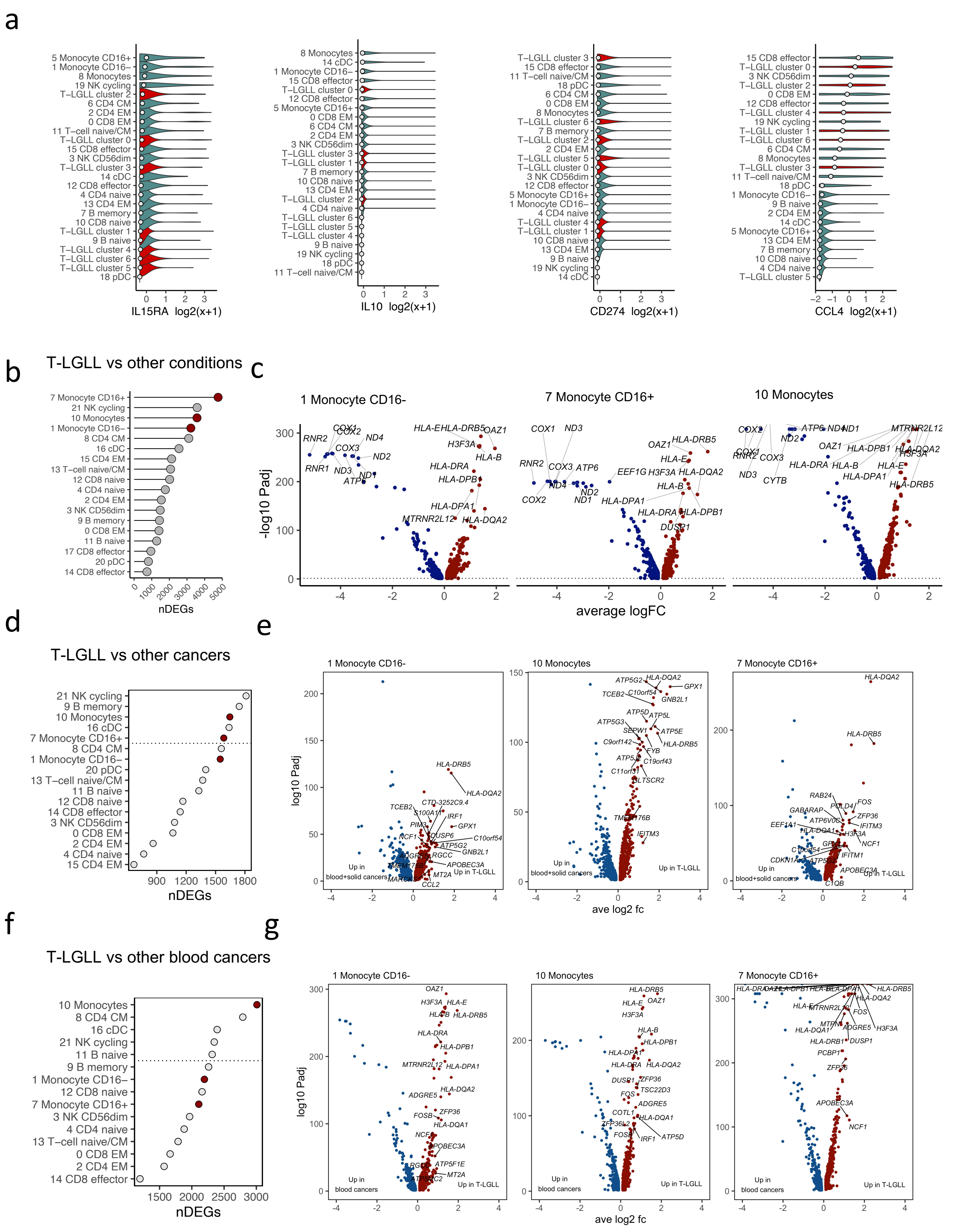

Supplementary Figure 15: Cytokine environment in T-LGLL in comparison to other cancers

a) Expression of *IL15RA*, *IL10*, *CD274* (PD-L1), and *CCL4* in non-leukemic and leukemic cell types in T-LGLL, where the median is highlighted. T-LGLL clonotype phenotypes are shown in red while other clusters are shown in green. **b)** Number of differentially expressed genes (DEGs, P_{adj}<0.05, Bonferroni corrected t-test) in each cell cluster between patients with T-LGLL and other conditions (combined patients with other cancers [CLL *n*=4, CML *n*=4, NSCLC *n*=1, RCC *n*=2] and healthy controls [*n*=6]). Different monocyte clusters are highlighted in red. **c)** Differentially expressed genes (P_{adj}<0.05, Bonferroni corrected two-sided t-test) between different monocyte clusters between patients with T-LGLL samples and patients with other cancers. **d)** Similar plot as in panel B but from patients with T-LGLL and patients with other cancers (CLL, CML, NSCLC, RCC). **e)** Similar plot as in panel C but from patients with T-LGLL and patients with other cancers (CLL, CML, NSCLC, RCC). **f)** Similar plot as in panel B but from patients with T-LGLL and patients with other blood cancers (CLL, CML). **g)** Similar plot as in panel C but from patients with T-LGLL and patients with other blood cancers (CLL, CML).

Supplementary Figure 16

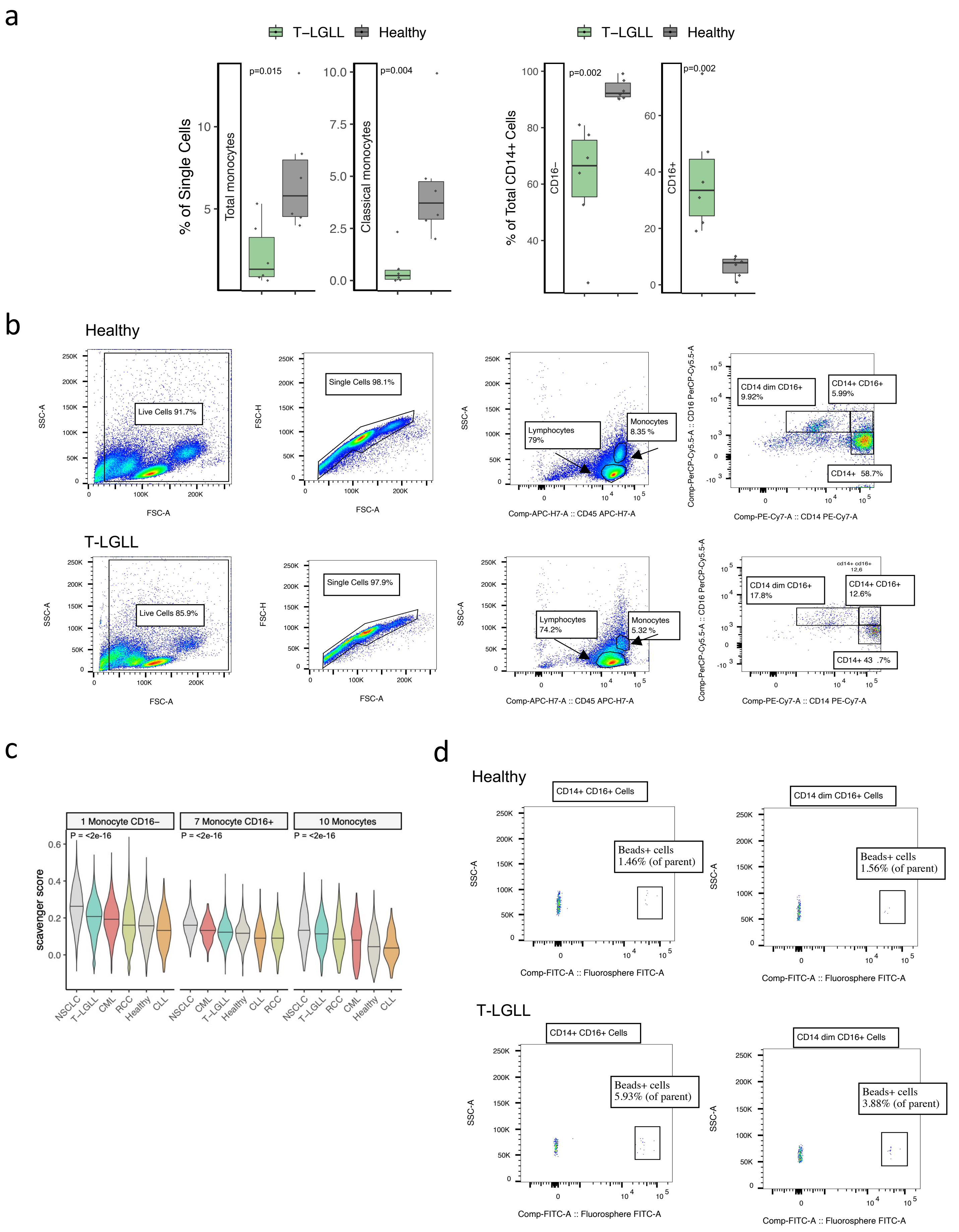

Supplementary Figure 16: Altered distribution of different monocytes subsets in T-LGLL

a) Left: The proportion of total and classical CD14+ monocytes as percentage of total single cells in flow cytometry data where the T-LGLL clone is not removed in patients with T-LGLL (*n*=6) and in healthy (*n*=6). Right: The proportion of CD16+ and CD16- monocytes as a percentage of total CD14+ cells in flow cytometry. *P*-values were calculated with two-sided Mann-Whitney test. **b)** Representative gating strategy for different monocyte populations with CD45 (APC-H7), CD14 (Pe-Cy7), and CD16 (PerCP-Cy5.5) in the validation cohort profiled with flow cytometry. **c)** Scavenger receptor score of different monocyte clusters in T-LGLL and in comparison to other conditions (CLL *n*=4, CML *n*=4, NSCLC *n*=1, RCC *n*=2, healthy *n*=6). *P*-values were calculated with two-sided Kruskal-Wallis test. **d)** Representative gating strategy for cells adhered to FluoSpere-beads (FITC) -stained cells in flow cytometry experiments in different monocyte populations in the validation cohort profiled with flow cytometry.

Supplementary Figure 17

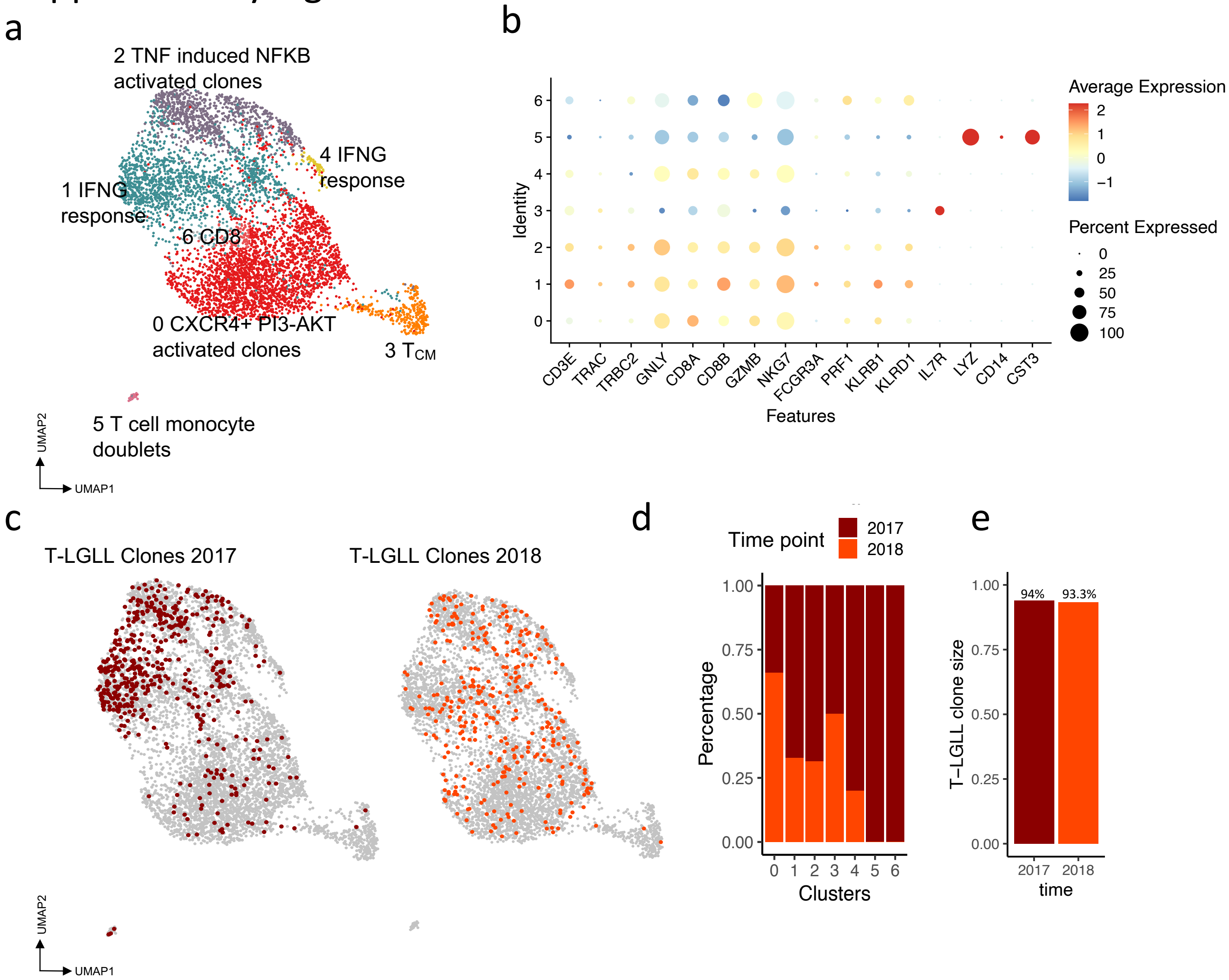

Supplementary Figure 17: The immune independent mutated *STAT3* clone is stable between time points

a) UMAP representation of the CD8+ T-cells from patient 2 from two different time points. **b)** Expression of canonical markers used to annotate the clusters. The dot size correlates with the number of cells expressing a given gene while the color denotes the gene expression as Z-values. **c)** *STAT3* mutated T-LGLL clone highlighted in the UMAPs in different time points. **d)** Proportion of cells belonging to different clusters in different time points. **e)** Size of the mutated *STAT3* T-LGLL clone in different time points.
