# Supplementary material for: Single-cell characterization of leukemic and non-leukemic immune repertoires in CD8+ T-cell large granular lymphocytic leukemia
Source: Nat Commun. 2022 Apr 11;13:1981. doi: 10.1038/s41467-022-29173-z (PMC9001660; doi:10.1038/s41467-022-29173-z)
Supplement: Supplementary file 3 — Description of Additional Supplementary Files [file 41467_2022_29173_MOESM3_ESM.pdf]

Description of Additional Supplementary Files

File Name: Supplementary Data 1
Description: Patient details from scRNA+TCRαβ-seq, bulk-RNA-seq, TCRβ-seq, flow cytometry, and published data as well as primer seqeunces

File Name: Supplementary Data 2
Description: Differentially expressed genes, pathways, cell abundances, and plasma cytokines

File Name: Supplementary Data 3
Description: Profiled and gathered T-LGLL clonotypes from publications and their analysis results

File Name: Supplementary Data 4
Description: GLIPH2 results for clones with antigen drive
