# Supplementary material for: Single-cell characterization of leukemic and non-leukemic immune repertoires in CD8+ T-cell large granular lymphocytic leukemia
Source: Nat Commun. 2022 Apr 11;13:1981. doi: 10.1038/s41467-022-29173-z (PMC9001660; doi:10.1038/s41467-022-29173-z)
Supplement: Supplementary file 8 — Reporting Summary [file 41467_2022_29173_MOESM8_ESM.pdf]

nature portfolio

Corresponding author(s): Mustjoki Satu

Last updated by author(s): 29.11.2021

Reporting Summary

Nature Portfolio wishes to improve the reproducibility of the work that we publish. This form provides structure for consistency and transparency in reporting. For further information on Nature Portfolio policies, see our Editorial Policies and the Editorial Policy Checklist.

Statistics

For all statistical analyses, confirm that the following items are present in the figure legend, table legend, main text, or Methods section.

n/a	Confirmed	
☐	☒	The exact sample size (*n*) for each experimental group/condition, given as a discrete number and unit of measurement
☐	☒	A statement on whether measurements were taken from distinct samples or whether the same sample was measured repeatedly
☐	☒	The statistical test(s) used AND whether they are one- or two-sided<br>*Only common tests should be described solely by name; describe more complex techniques in the Methods section.*
☐	☒	A description of all covariates tested
☐	☒	A description of any assumptions or corrections, such as tests of normality and adjustment for multiple comparisons
☐	☒	A full description of the statistical parameters including central tendency (e.g. means) or other basic estimates (e.g. regression coefficient) AND variation (e.g. standard deviation) or associated estimates of uncertainty (e.g. confidence intervals)
☐	☒	For null hypothesis testing, the test statistic (e.g. *F*, *t*, *r*) with confidence intervals, effect sizes, degrees of freedom and *P* value noted<br>*Give P values as exact values whenever suitable.*
☒	☐	For Bayesian analysis, information on the choice of priors and Markov chain Monte Carlo settings
☐	☒	For hierarchical and complex designs, identification of the appropriate level for tests and full reporting of outcomes
☒	☐	Estimates of effect sizes (e.g. Cohen's *d*, Pearson's *r*), indicating how they were calculated

Our web collection on statistics for biologists contains articles on many of the points above.

Software and code

Policy information about availability of computer code

Data collection

All custom scripts made for scRNA-seq, TCRαβ-seq and healthy data are public to everyone and the codes to replicate major findings can be found at the projects github page: https://github.com/janihuuh/cd8_tlgll_manu

Data analysis

Programmes
• R (4.0.2)
• Python (3.7.4)
• BD FACS Suite (1.0.6)
• FlowJo software Tools (ver 10.7)
• Seurat (ver 3.0.0) (R)
• SingleR (ver 1.2.4) (R)
• Slingshot (ver 1.1.4) (R)
• ClusterProfiler (3.16.0) (R)
• SCENIC (ver 1.2.4) (R)
• edgeR (3.3.3) (R)
• scVI (ver 0.5.0) (python)

• TCRGP (ver 1.0.0) (python)

• Cell Ranger (ver 2.1.1) (command-line)
• CellPhoneDB (ver 2.0.0) (command line)
• Vartrix (ver 1.1.0) (command line)
• MiXCR (ver 3.0.13) (command line)
• VDJtools (ver 1.2.1) (command line)

• GLIPH2 (1.0.0) (command line)

Instructions
1. System requirements: Most of the analyses have been done on 2,5 GHz 14-Core Intel Xeon W, iMac Pro with 128 GB 2666 MHz DDR4. The more computationally demanding analyses were performed with the computational resources provided by the Aalto Univeristy Science-IT project.
2. Installation guide for the software: For this manuscript, we have not generated any custom-made or in-house software. All the softwares and tools used have been cited in the paper and have their own installation guidelines.
3. Demo: scRNA+TCRab-seq (1 T-LGLL and 1 healthy) and TCRb-seq data (1 T-LGLL and 1 healthy) can be found in the provided zip-file. The demo file contains only n=1 for both groups due to the limits in the demo data.
4. Source codes for figures are available in Source_data.xlsx file.

For manuscripts utilizing custom algorithms or software that are central to the research but not yet described in published literature, software must be made available to editors and reviewers. We strongly encourage code deposition in a community repository (e.g. GitHub). See the Nature Portfolio guidelines for submitting code & software for further information.

Data

Policy information about availability of data

All manuscripts must include a data availability statement. This statement should provide the following information, where applicable:

- Accession codes, unique identifiers, or web links for publicly available datasets
- A description of any restrictions on data availability
- For clinical datasets or third party data, please ensure that the statement adheres to our policy

Data Availability statement: The scRNA-sequencing data and bulk-RNA-sequencing data for both the T-LGLL and healthy samples data generated in this study have been deposited in the EGA database under accession code EGAS00001005297[https://ega-archive.org/studies/EGAS00001005297]. The processed scRNA-sequencing data are available at ArrayExpression under accession code E-MTAB-11170[https://www.ebi.ac.uk/arrayexpress/experiments/E-MTAB-11170] and the TCRαβ-sequencing data, TCRβ-sequencing data, and Seurat-objects are available at Zenodo under DOI: 10.5281/zenodo.4739231. The publicly available scRNA +TCRαβ-sequencing and TCRβ-sequencing data used in this study are listed in Supplementary Table 1. Source data are provided with this manuscript

Field-specific reporting

Please select the one below that is the best fit for your research. If you are not sure, read the appropriate sections before making your selection.

☒ Life sciences ☐ Behavioural & social sciences ☐ Ecological, evolutionary & environmental sciences

For a reference copy of the document with all sections, see nature.com/documents/nr-reporting-summary-flat.pdf

Life sciences study design

All studies must disclose on these points even when the disclosure is negative.

Sample size	Sample size calculations were not performed. We performed sc-RNA+TCRab-seq from all available samples at the time of study initiation. Similarly, all available sample material was used for bulk-RNA-seq and for TCR-beta-seq. For functional validations we similarly used all available suitable samples (considering clone size and STAT3 mutation status) at the time of study initiation.<br>We concluded that these group sizes were sufficiently large from previous experience (Kasanen et al, Cancer Immunology Immunotherapy, 2020, PMID: 32036449)
Data exclusions	No data was excluded
Replication	For flow-cytometry validations, all assays were performed with at least 6 biological replicates(= individual samples) per group. We have reported all acquired data and subsequently performed experiments was concordant.
Randomization	Our study compared T-LGLL patients and healthy controls. We relied on the clinical diagnosis set by an experienced hematologist. Further randomization is not relevant for this study.
Blinding	In all flow-cytometry sortings, prior to single cell sequencing, identical gates were used for patients and controls. This was done to ensure all samples are objectively treated without any bias. All investigators involved in subsequent data acquisition were blinded to the samples identity. The functional flow assays were performed by a technician who was blinded to the study.<br>Data analysis investigators were not blinded for samples identity and group annotation. This was not relevant for the main objectives of the study as data analysis was performed using computational methods to ensure objective interpretation of the data.

Reporting for specific materials, systems and methods

We require information from authors about some types of materials, experimental systems and methods used in many studies. Here, indicate whether each material, system or method listed is relevant to your study. If you are not sure if a list item applies to your research, read the appropriate section before selecting a response.

Materials & experimental systems

n/a	Involved in the study	
☐	☒	Antibodies
☒	☐	Eukaryotic cell lines
☒	☐	Palaeontology and archaeology
☒	☐	Animals and other organisms
☐	☒	Human research participants
☒	☐	Clinical data
☒	☐	Dual use research of concern

Methods

n/a	Involved in the study	
☒	☐	ChIP-seq
☐	☒	Flow cytometry
☒	☐	MRI-based neuroimaging

Antibodies

Antibodies used

Antibodies, flurochormes, the clones, Catalog numbers and Company-

anti-CD3 PeCy7 (Clone: SK7, Cat#: 557851, Lot#: 8037645, BD Biosciences)
anti-CD4 PerCP (Clone: SK3, Cat#: 345770, Lot#: 6281605, BD Biosciences)
anti-CD8 PerCP (Clone: SK1, Cat#: 345774, Lot#: 82152, BD Biosciences)
anti-CD45+ APC H7 (Clone: 2D1, Cat#: 560178 BD Biosciences)

anti-CD3 APC (Clone: UCHT1, Cat#: 555335, BD Biosciences)
anti-CD49d (Clone: L25 BD, Cat#: 340976, BD Biosciences)
anti-CD28 (Clone: L293, Cat. 340975, BD Biosciences)
anti-CD107a FITC (Clone: H4A3, Cat. 555800 , BD Biosciences)
anti- CD107b FITC (Clone:H4B4, Cat. 555804, BD Biosciences)

anti-CD57 PE (Clone:NK-1 Cat#: 560844, BD Biosciences)
anti-CD8 PE-CY7 (Clone:SK1 , Cat#: 335822, BD Biosciences)
anti-CD45 V500 (Clone:2D1 , Cat#: 655873, BD Biosciences)
anti-TNF V450 (Clone: MAb11, Cat#: 561311, BD Biosciences)
anti-FNG V450 (Clone:B27 , Cat#: 560371, BD Biosciences)

anti-perforin 1 PerCP-Cy5.5 (Clone: δG9, Cat#: 563762, BD Biosciences)
anti-granzyme A AlexaFluor700 (Clone:CB9, Cat#: 507210, Biolegend)
anti-granzyme B AlexaFluor700 (Clone:GB11, Cat#: 561016, 560213, BD Biosciences)

CD14 Pe-Cy7 (Clone:MϕP9, Cat#: 562698, BD Biosciences)
CD16 PerCP-Cy5.5 (Clone:3G8, Cat#: 560717, BD Biosciences)
CD45 APC-H7 (Clone:2D1, Cat#: 641417, BD Biosciences)

TCR Vbeta antibodies (IOTest® Beta Mark TCR Vbeta Repertoire Kit, Cat#: IM3497, Lot#: 66, Beckman Coulter)
The antibodies were not diluted and used as per the manufacturers guidelines (shown on their websites).

Validation

All antibodies used are commercially available and formally validated by the vendors. Validation information for all antibodies is reported in technical data sheets available on vendors' homepage.

Human research participants

Policy information about studies involving human research participants

Population characteristics

Extensive clinical data about the patients, including treatment information, co-morbidities and blood counts, have been provided in Supplementary Table 1 (sheet: scRNAseq and flow). For each experiment we selected age and sex matched healthy controls. As we compared the patients to healthy controls, controlling for treatment history or previous diagnosis was not relevant to this study.

sc-RNA-seq+TCRab and functional validations

Patient	Assay	Genotype	age at dg	Gender
Pt 1	scRNAseq	Y640F 21%	58	Female
Pt 1 (timepoint 2)	scRNAseq	Y640F 2%	58	Female
Pt 2	scRNAseq	D661Y 45%	55	Male
Pt 2 (timepoint 2)	scRNAseq	D661Y 50%	55	Male
Pt 3	scRNAseq	Y640F 6%	53	Female
Pt 4	scRNAseq	Y640F 25%	67	Female
Pt 5	scRNAseq	Y640F 7%	74	Female
Pt 6	scRNAseq	Wild type	75	Male
Pt 7	scRNAseq	Y640F 39%	65	Female
Pt 8	scRNAseq	S614R 39% D170H 1.5%	84	Male
Pt 9	scRNAseq	D661Y 12%, Y640F 3%, N647I 0.02%, I659L 0.01%	36	Male

Sample	Assay	Genotype	age at dg	Gender
Pt 10	flow validation cohort	Tyr640Phe	42	Female
Pt 11	flow validation cohort	Wild type	69	Female
Pt 12	flow validation cohort	Wild type	69	Male
Pt 13	flow validation cohort	c1981G>T	51	Female
Pt 14	flow validation cohort	D566N 33.52%, K290N 33% (2011)	33	Female
Pt 15	flow validation cohort	Y640F 50%, E398K 2.5% (2012)	44	Male
HC 1	scRNAseq and flow validation cohort	Wild type	52	Male
HC 2	scRNAseq and flow validation cohort	Wild type	58	Male
HC 3	scRNAseq and flow validation cohort	Wild type	56	Male
HC 4	scRNAseq and flow validation cohort	Wild type	56	Female
HC 5	scRNAseq and flow validation cohort	Wild type	63	Female
HC 6	scRNAseq and flow validation cohort	Wild type	55	Female

Bulk-RNA-seq

Sample	Genotype	Gender
Healthy 1	WT	F
Healthy 2	WT	M
Healthy 3	WT	M
Healthy 4	WT	M
Healthy 5	WT	F
Healthy 6	WT	M
Healthy 7	WT	Unknown
Healthy 8	WT	Unknown
T-LGLL 1 CD8+ T-LGLL	D661Y	M
T-LGLL 2 CD8+ T-LGLL	Y640F	F
T-LGLL 3 CD8+ T-LGLL	Y640F	M
T-LGLL 4 CD8+ T-LGLL	D661H	F
T-LGLL 5 CD8+ T-LGLL	D661Y	F
T-LGLL 6 CD8+ T-LGLL	Y640F	M
T-LGLL 7 CD8+ T-LGLL	D661V	F
T-LGLL 8 CD8+ T-LGLL	D661Y	M
T-LGLL 9 CD8+ T-LGLL	D661V, Y640F	M
T-LGLL 10 CD8+ T-LGLL	Y640F F	
T-LGLL 11 CD8+ T-LGLL	WT	M
T-LGLL 12 CD8+ T-LGLL	WT	F
T-LGLL 13 CD8+ T-LGLL	WT	F
T-LGLL 14 CD8+ T-LGLL	WT	M
T-LGLL 16 CD8+ T-LGLL	WT	M

plasma cytokines

Name Type Gender Age (at Sampling)
Pt 1 T-LGLL Male 61
Pt 2 T-LGLL Female 53
Pt 3 T-LGLL Female 54
Pt 4 T-LGLL Male 61
Pt 5 T-LGLL Male 59
Pt 6 T-LGLL Female 65
Pt 7 T-LGLL Female 75
Pt 8 T-LGLL Female 67
Pt 9 T-LGLL Female 64
HC1 Healthy Male 49
HC2 Healthy Female 43
HC3 Healthy Female 36
HC4 Healthy Female 24
HC5 Healthy Female 29
HC6 Healthy Female 59
HC7 Healthy Male 65
HC8 Healthy Female 28

Recruitment

Samples from T-LGLL patients were collected at the Helsinki University Hospital Comprehensive Cancer Center (Finland), Cleveland Clinic (USA), University Clinic of Cologne (Germany), University Hospital of Padova (Italy), and Shinshu University School of Medicine (Japan). Patients were recruited following routine diagnostic procedures.
We used all available samples and recruitment was done by hematologists who were not directly involved in the study design.

Ethics oversight

The study was approved by local ethical committees. Written informed consent was received from all patients and the study was conducted in accordance with the Declaration of Helsinki.
The following institutes ethically approved the protocol: Hematology Research Unit Helsinki, University of Helsinki and Helsinki University Hospital Comprehensive Cancer Center, Helsinki, Finland; Translational Hematology and Oncology Department, Taussig Cancer Center, Cleveland Clinic, Cleveland, OH, USA; Department I of Internal Medicine, Center for Integrated Oncology (CIO), Aachen-Bonn-Cologne-Duesseldorf, University of Cologne (UoC), Cologne, Germany; Clinic of

Hematology and Cellular Therapy, University of Leipzig, Leipzig, Germany; Department of Medicine (DIMED), Hematology and Clinical Immunology Branch, Padova University School of Medicine, Italy;
Department of Biomedical Laboratory Sciences, Shinshu University School of Medicine, Matsumoto, Japan; Division of Hematology, Department of Internal Medicine, Shinshu University School of Medicine, Matsumoto, Japan;
and 13Division of Hematology/Oncology, Department of Medicine, UVA Cancer Center, University of Virginia, Charlottesville, VA, USA.

Note that full information on the approval of the study protocol must also be provided in the manuscript.

Flow Cytometry

Plots

Confirm that:

☒ The axis labels state the marker and fluorochrome used (e.g. CD4-FITC).

☒ The axis scales are clearly visible. Include numbers along axes only for bottom left plot of group (a 'group' is an analysis of identical markers).

☒ All plots are contour plots with outliers or pseudocolor plots.

☒ A numerical value for number of cells or percentage (with statistics) is provided.

Methodology

Sample preparation

Peripheral blood was collected into EDTA tubes. Mononuclear cells were separated using density gradient centrifugation with Ficoll-PaqueTM PLUA (GE Healthcare)

Instrument

FACSVerse (BD Biosciences) was used to acquire data.

Software

Collection: BD FACSuite Software (version 1.0.6)
Analysis: Flowjo (Version 10.6.1)

Cell population abundance

One million stained cells were suspended stained per 300 uL phosphate buffered saline for flow sorting. Purity of the sorted cells (FACs Aria II, BD Biosciences) was confirmed using the same instrument.

Gating strategy

For all flow sorting and analysis, the first step was to select live cells from FSC-A versus SSC-A scatter, everything below 30K in the FSC-A axis were removed as debirs/dead cells. This was followed by selection of single cells (doublet removal) from FSC-A versus FSC-H and everything below 30K in the FSC-A axis were removed as debirs/dead cells. Unstained cells were used to set the boundaries between negative and positive populations.
Applied exemplifying gating strategy is described in detail in the supplementary figures 1a, 6a, 13e-g,16b and 16d .

☒ Tick this box to confirm that a figure exemplifying the gating strategy is provided in the Supplementary Information.
